# A real-world safety surveillance study of aducanumab through the FDA adverse event reporting system

**DOI:** 10.3389/fphar.2025.1522058

**Published:** 2025-03-13

**Authors:** Jingjing Huang, Xiaohong Long, Chunyong Chen

**Affiliations:** ^1^ Cardiac Intensive Care Unit, The First Affiliated Hospital of Guangxi Medical University, Nanning, Guangxi, China; ^2^ Department of Neurology, The First Affiliated Hospital of Guangxi Medical University, Nanning, Guangxi, China

**Keywords:** food drug administration adverse event reporting system (FAERS), aducanumab, monoclonal antibody selectively target, real-world study, adverse events

## Abstract

**Background:**

Alzheimer’s disease poses a major public health challenge, with aducanumab’s approval in 2021 as the first disease-modifying therapy raising important safety considerations. This study analyzed the Food Drug Administration Adverse Event Reporting System (FAERS) database to evaluate aducanumab’s real-world safety profile and identify potential risk factors.

**Methods:**

We conducted a comprehensive pharmacovigilance study using the FAERS database from January 2004 to June 2024, analyzing 510 aducanumab-associated reports from integrated databases containing over 18 million demographic records and 66 million drug records. Safety signals were evaluated using four complementary disproportionality methods: Reporting Odds Ratio (ROR), Proportional Reporting Ratio (PRR), Bayesian Confidence Propagation Neural Network (BCPNN), and Multi-item Gamma Poisson Shrinker (MGPS). Analyses were stratified by age and sex, with adverse events examined at both System Organ Class (SOC) and Preferred Term (PT) levels using SAS 9.4.

**Results:**

Among 510 aducanumab-associated adverse event reports, predominantly from elderly patients (55.49% aged ≥65 years), nervous system disorders were the most frequent (53.24%, n = 583). Amyloid related imaging abnormality-oedema/effusion (ARIA-E) and Amyloid related imaging abnormality-microhaemorrhages and haemosiderin deposits (ARIA-H) emerged as the most significant safety signals (ROR: 53,538.3 and 38,187.9, respectively). Sex-stratified analysis showed comparable safety profiles between males and females, with ARIA-E related events, ARIA-H related events, maintaining strong signals across all age groups, particularly in patients ≥75 years. The median time to adverse event onset was 146.0 days (IQR: 80.0–195.0). Temporal analysis revealed increasing signal strength for ARIA-related events from 2004–2024, with notable intensification during 2022–2023.

**Conclusion:**

Our real-world analysis identified ARIA-related events as the primary safety concern for aducanumab, typically occurring within 146 days of treatment initiation, with comparable safety profiles across sex but heightened risks in patients ≥75 years. These findings support aducanumab’s viability as a therapeutic option while emphasizing the critical importance of rigorous monitoring protocols, particularly for ARIA events during the first year of treatment.

## 1 Introduction

Alzheimer’s disease (AD) represents an unprecedented healthcare challenge, with 6.9 million Americans over 65 currently diagnosed. This neurological burden is projected to increase substantially, as epidemiological models predict 13.8 million cases by 2060 (2024 Alzheimer’s disease facts and figures, 2024). The devastating impact of AD extends beyond individual patients to families, healthcare systems, and societies, with global costs exceeding $1 trillion annually (Skaria, 2022).

Despite decades of research, therapeutic options for AD have remained limited, with most available treatments providing only symptomatic relief rather than addressing the underlying pathology (Passeri et al., 2022; van der Flier et al., 2023). The amyloid hypothesis, which posits that β-amyloid (Aβ) accumulation is central to AD pathogenesis, has driven drug development efforts, though many candidates have failed in clinical trials (Selkoe and Hardy, 2016; Wu et al., 2022).

A breakthrough occurred in 2021 with the FDA’s accelerated approval of aducanumab, the first disease-modifying therapy for AD (Tampi et al., 2021). This monoclonal antibody selectively targets and removes aggregated Aβ from the brain, representing a paradigm shift in AD treatment (Ali et al., 2022). The approval, while controversial, opened a new era in AD therapeutics and provided hope for millions of patients and their families.

However, the introduction of aducanumab into clinical practice has raised important safety considerations. The drug’s mechanism of action, involving the clearance of cerebral amyloid, can lead to Amyloid-Related Imaging Abnormalities (ARIA) and other potential adverse effects (Agarwal et al., 2023; Hampel et al., 2023). As real-world use expands, comprehensive understanding of its safety profile becomes increasingly crucial for optimal patient care (Knopman et al., 2021).

Clinical trials of aducanumab provide crucial foundational safety data through rigorous scientific protocols, even with the natural constraints of sample size, study duration and population representation (Padala and Yarns, 2022). The progression from controlled trials to real-world practice represents a natural evolution in drug development, bringing distinct challenges in how we gather and analyze clinical data (Galvin et al., 2024). Real-world evidence is essential for understanding the full spectrum of adverse events and identifying potential risk factors across different patient populations. Recent initiatives, such as the International Collaboration for Alzheimer’s Research and Evaluation in Alzheimer’s Disease (ICARE AD) study, demonstrate the ongoing efforts to gather real-world safety data, despite the inherent complexities of post-marketing surveillance (Heidebrink and Paulson, 2024). Current pharmacovigilance studies, while establishing preliminary insights, need continuous updating as new data become available (Ameen et al., 2024). The evolution of safety monitoring systems, supported by advancing analytical capabilities (Silva et al., 2024), will gradually enhance our understanding of aducanumab’s safety profile in routine clinical practice.

Our study addresses these knowledge gaps through a comprehensive analysis of the FDA Adverse Event Reporting System (FAERS) database, employing multiple complementary analytical approaches. While preclinical animal studies provided initial safety insights, their translational value has inherent limitations, highlighting the critical need for real-world safety assessments (Marshall et al., 2023). This investigation aims to characterize the safety profile of aducanumab in real-world settings, identify potential risk factors, and provide evidence-based guidance for clinical monitoring.

## 2 Materials and methods

### 2.1 Data source and study design

**FIGURE 1:**
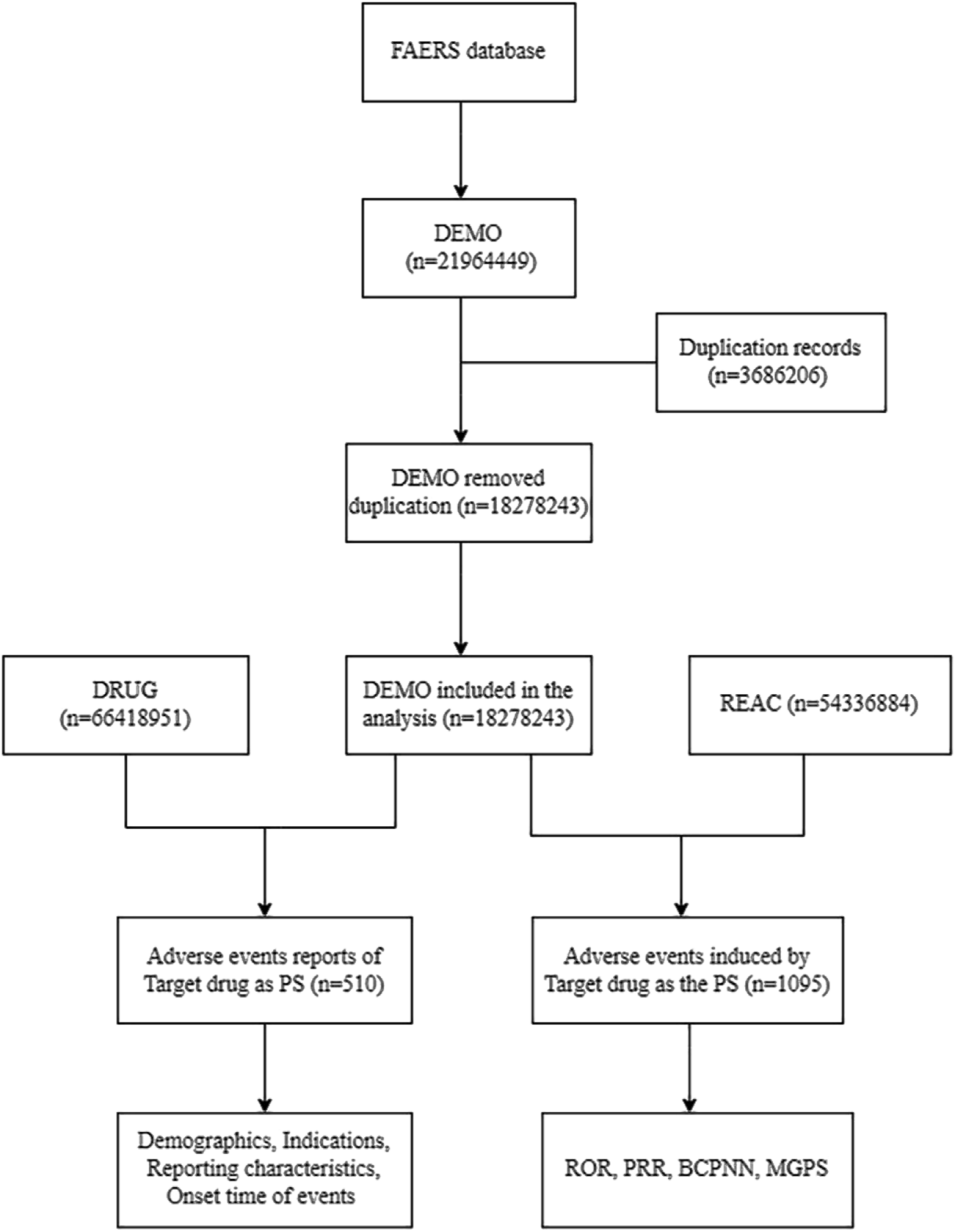
Flow diagram of data extraction and analysis process for aducanumab adverse events from the FAERS database. Abbreviation: BCPNN, Bayesian confidence propagation neural network; DEMO, Demographics; FAERS, FDA adverse event reporting system; MGPS, Multi-item gamma poisson shrinker; PRR, Proportional reporting ratio; PS, Primary suspect; REAC, Reactions; ROR, Reporting odds ratio.

We analyzed adverse event reports from the FAERS database (January 2004-June 2024) after deduplication, integrating DEMO (n = 18, 278, 243), DRUG (n = 66, 418, 951), and REAC (n = 54, 336, 884) databases (Figure 1). The analysis focused on aducanumab as the primary suspect drug, examining both demographic patterns (n = 510 reports) and safety signals (n = 1,095 events) using four disproportionality methods of Proportional Reporting Ratio (PRR); Bayesian Confidence Propagation Neural Network (BCPNN) using Information Component (IC); Multi-item Gamma Poisson Shrinker (MGPS) using Empirical Bayes Geometric Mean (EBGM).

### 2.2 Signal detection analysis

For robust signal detection, we employed four complementary analytical methods (Supplementary Table S1): Reporting Odds Ratio (ROR) with 95% confidence intervals (CI), PRR, BCPNN, and MGPS. Each method provided unique statistical perspectives to ensure comprehensive safety signal evaluation. Each method provided unique statistical insights, confirming the robustness of our findings. The safety signal was considered significant when meeting all the following criteria: Case count ≥3, Lower bound of ROR 95% CI > 1, PRR ≥2 with χ^2^ ≥ 4, IC-2SD > 0, EBGM05 > 2.

### 2.3 Statistical analysis

Adverse events were analyzed at both System Organ Class (SOC) and Preferred Term (PT) levels. We performed stratified analyses by age (45–64, 65–74, and ≥75 years) and sex. Time-to-onset analysis was conducted using cumulative distribution analysis. Descriptive statistics were presented as frequencies and percentages for categorical variables, and means (SD) or medians (IQR) for continuous variables. To detect potential safety signals, we employed multiple disproportionality analyses. These included the reporting odds ratio (ROR) with its 95% confidence interval, the proportional reporting ratio (PRR) assessed through chi-square testing, the Information Component (IC) evaluated with a two-standard-deviation threshold (IC-2SD), and the Empirical Bayes Geometric Mean (EBGM) with its 95% confidence interval. All analyses were performed using SAS 9.4 (Statistical Analysis System, version 9.4; SAS Institute Inc., Cary, NC, United States).

## 3 Results

### 3.1 Demographic and clinical characteristics

During the surveillance period from January 2004 to June 2024, 510 reports of adverse events associated with aducanumab were documented in the Food Drug Administration Adverse Event Reporting System (FAERS) database. Table 1 presents the demographic and clinical characteristics of these cases. The sex distribution was relatively balanced, with 49.80% (n = 254) being female and 44.51% (n = 227) male, while 5.69% (n = 29) of reports had unknown sex status. Age analysis revealed 32.55% (n = 166) aged 75 years or older, and 22.94% (n = 117) aged between 65 and 74 years. Temporal distribution highlighted a concentration of reports in recent years, particularly in 2022 (43.92%, n = 224) and 2023 (39.02%, n = 199). The primary sources of these reports were consumers (41.57%, n = 212), followed by physicians (34.31%, n = 175) and pharmacists (23.53%, n = 120). Geographically, the reports were overwhelmingly from the United States (92.75%, n = 473), with Japan (1.96%, n = 10) and Canada (0.98%, n = 5) also contributing.

**TABLE 1:** Clinical and demographic overview of FAERS-reported aducanumab cases (2004–2024).

Characteristics	Case number, n (%)
Sex	
Female	254 (49.80)
Male	227 (44.51)
Unknown	29 (5.69)
Age	
<18	0 (0.00)
18–44	0 (0.00)
45–64	29 (5.69)
65–74	117 (22.94)
≥75	166 (32.55)
Unknown	198 (38.82)
Reporting year	
2016	18 (3.53)
2018	1 (0.20)
2019	2 (0.39)
2021	18 (3.53)
2022	224 (43.92)
2023	199 (39.02)
2024	48 (9.41)
Reported person	
Consumer	212 (41.57)
Not specified	3 (0.59)
Pharmacist	120 (23.53)
Physician	175 (34.31)
Reported Countries (Top Five)	
United States	473 (92.75)
Japan	10 (1.96)
Canada	5 (0.98)
France	4 (0.78)
Switzerland	4 (0.78)
AE severity	
Serious	258 (50.59)
Non-Serious	252 (49.41)
Outcome	
Life-threatening	8 (1.57)
Hospitalization - initial or prolonged	121 (23.73)
Disability	2 (0.39)
Death	29 (5.69)
Congenital anomaly	0 (0.00)
Required intervention to Prevent permanent Impairment/damage	2 (0.39)
Other	135 (26.47)
Time to onset of SG related AEs	
0–30 days	25 (4.90)
31–60 days	18 (3.53)
61–90 days	21 (4.12)
91–120 days	33 (6.47)
121–150 days	23 (4.51)
151–180 days	39 (7.65)
181–360 days	43 (8.43)
>360 days	15 (2.94)
Weight (Kg)	
N (Missing)	170 (340)
Mean (SD)	71.49 (16.36)
Median (Q1,Q3)	69.62 (60.80,80.30)
Min, Max	34.20,132.00

Abbreviations: AE, adverse event; CI, confidence interval; FAERS, FDA, adverse event reporting system; IQR, interquartile range; Max, Maximum; Min, Minimum; Q1, first quartile; Q3, third quartile; SD, standard deviation; SG, suspected group; USA, United States.

### 3.2 Adverse event distribution by system organ class

**FIGURE 2:**
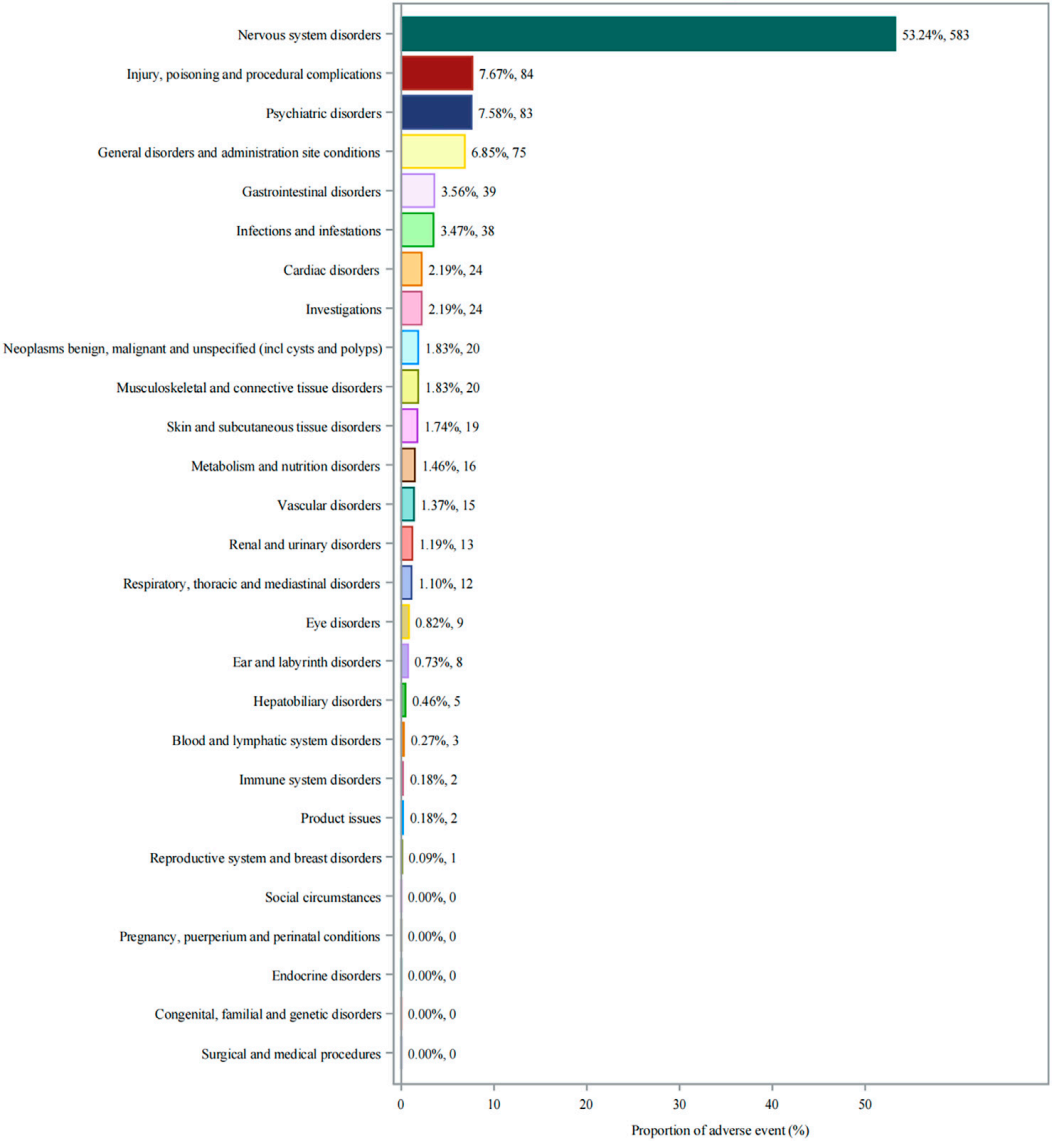
System organ class distribution of adverse events associated with aducanumab. Abbreviations: AE, Adverse event; CNS, Central nervous system; FAERS, FDA adverse event reporting system; SOC, System organ class.

Analysis of adverse events by SOC revealed that nervous system disorders were predominant, accounting for 53.24% (n = 583) of all reported events (Figure 2). This was followed by injury, poisoning and procedural complications (7.67%, n = 84) and psychiatric disorders (7.58%, n = 83). General disorders and administration site conditions (6.85%, n = 75) and gastrointestinal disorders (3.56%, n = 39) were also notably represented disorders (3.56%, n = 39).

### 3.3 Disproportionality analysis at SOC level

As detailed in Table 2, disproportionality analysis identified 22 system organ classes (SOCs) of adverse events. Nervous system disorders were the most frequently reported, comprising 352 cases with significant disproportionality (ROR of 10.82, PRR of 4.04), followed by injury, poisoning, and procedural complications (n = 63), and psychiatric disorders (n = 58). Psychiatric disorders and ear and labyrinth disorders, despite showing notable signals, did not reach statistical significance.

**TABLE 2:** Disproportionality analysis of aducanumab-related adverse events by system organ class in FAERS.

SOC	Case number (n)	ROR (95% CI)	PRR (χ2)	IC (IC-2SD)	EBGM
Nervous system disorders	352	10.82 (8.97, 13.05)	4.04 (971.65)	2.01 (1.80)	4.04 (3.35)
Injury, poisoning and procedural complications	63	0.24 (0.19, 0.32)	0.34 (129.25)	−1.57 (−1.94)	0.34 (0.26)
Psychiatric disorders	58	1.13 (0.86, 1.48)	1.11 (0.73)	0.15 (−0.25)	1.11 (0.85)
General disorders and administration site conditions	53	0.39 (0.29, 0.52)	0.45 (45.09)	−1.14 (−1.54)	0.45 (0.34)
Gastrointestinal disorders	36	0.59 (0.42, 0.83)	0.62 (9.55)	−0.69 (−1.17)	0.62 (0.44)
Infections and infestations	28	0.31 (0.21, 0.46)	0.35 (39.51)	−1.51 (−2.03)	0.35 (0.24)
Investigations	20	0.66 (0.42, 1.04)	0.68 (3.27)	−0.56 (−1.19)	0.68 (0.43)
Cardiac disorders	20	0.59 (0.38, 0.93)	0.61 (5.38)	−0.72 (−1.33)	0.61 (0.39)
Musculoskeletal and connective tissue disorders	18	0.26 (0.16, 0.42)	0.29 (36.00)	−1.79 (−2.42)	0.29 (0.18)
Neoplasms benign, malignant and unspecified (incl cysts and polyps)	16	0.27 (0.16, 0.44)	0.29 (30.60)	−1.77 (−2.42)	0.29 (0.18)
Skin and subcutaneous tissue disorders	15	0.54 (0.33, 0.91)	0.56 (5.54)	−0.84 (−1.54)	0.56 (0.33)
Metabolism and nutrition disorders	15	0.28 (0.17, 0.47)	0.30 (26.61)	−1.72 (−2.39)	0.30 (0.18)
Vascular disorders	15	0.51 (0.30, 0.85)	0.52 (6.93)	−0.94 (−1.63)	0.52 (0.31)
Renal and urinary disorders	13	0.58 (0.34, 1.01)	0.60 (3.74)	−0.75 (−1.49)	0.60 (0.34)
Respiratory, thoracic and mediastinal disorders	10	0.19 (0.10, 0.35)	0.21 (34.04)	−2.28 (−3.06)	0.21 (0.11)
Eye disorders	9	0.43 (0.22, 0.84)	0.44 (6.57)	−1.18 (−2.02)	0.44 (0.23)
Ear and labyrinth disorders	7	1.22 (0.58, 2.58)	1.22 (0.28)	0.29 (−0.78)	1.22 (0.58)
Hepatobiliary disorders	3	0.14 (0.05, 0.45)	0.15 (15.26)	−2.75 (−3.86)	0.15 (0.05)
Blood and lymphatic system disorders	3	0.27 (0.09, 0.84)	0.28 (5.86)	−1.86 (-3.02)	0.28 (0.09)
Product issues	2	0.12 (0.03, 0.5)	0.13 (12.28)	−2.97 (−4.15)	0.13 (0.03)
Immune system disorders	1	0.05 (0.01, 0.33)	0.05 (19.89)	−4.39 (−5.51)	0.05 (0.01)
Reproductive system and breast disorders	1	0.10 (0.01, 0.68)	0.10 (8.58)	−3.37 (−4.55)	0.10 (0.01)

Abbreviations: CI, confidence interval; EBGM, empirical bayes geometric mean; FAERS, FDA, adverse event reporting system; IC, information component; IC-2SD, information component minus two standard deviations; PRR, proportional reporting ratio; ROR, reporting odds ratio; SOC, system organ class.

### 3.4 Specific adverse event signals and profile

**FIGURE 3:**
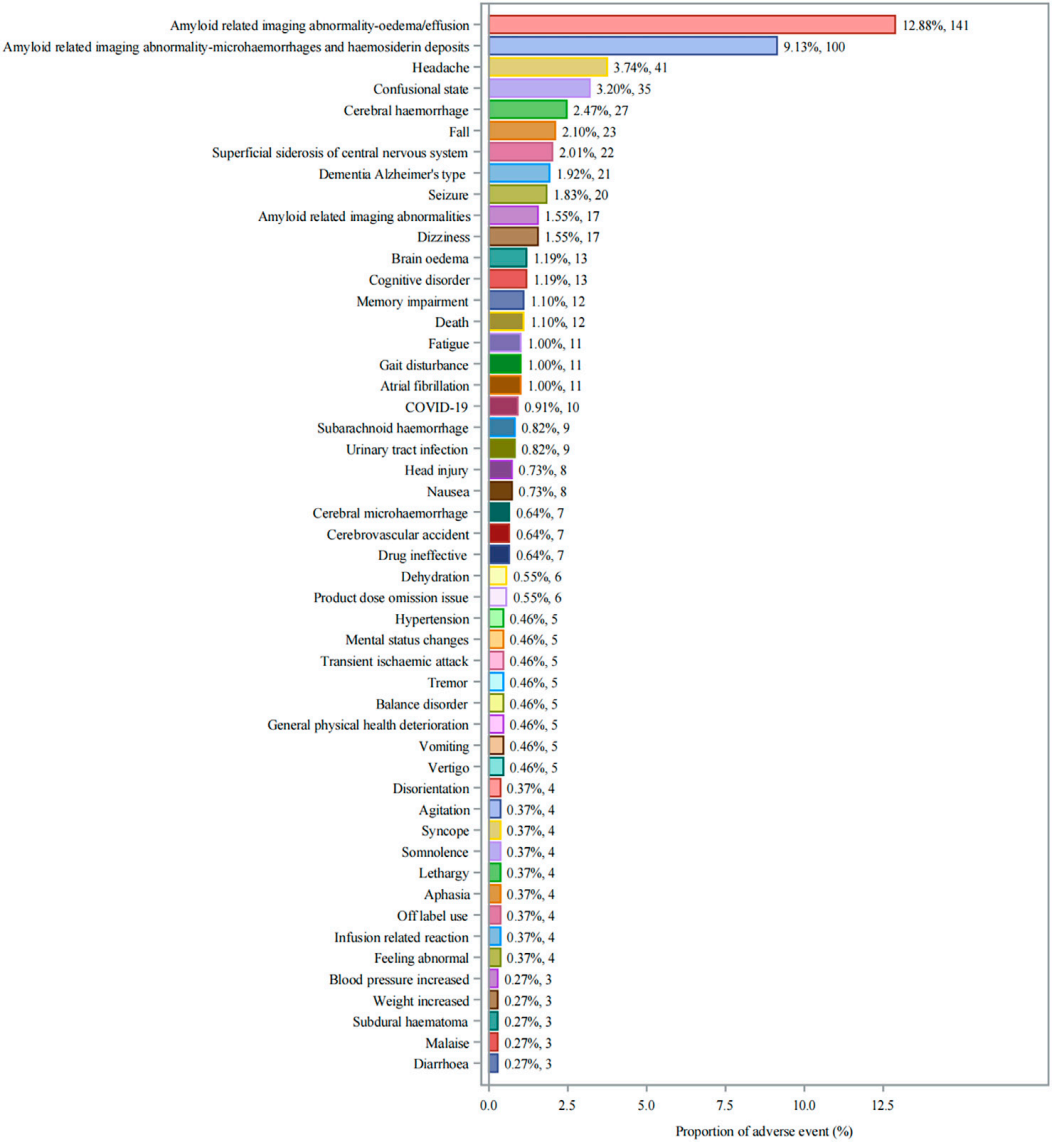
Distribution of Adverse Events Associated with Aducanumab: Analysis of Case Numbers and Proportions. Frequency distribution of adverse events reported with aducanumab use. The x-axis shows the proportion of adverse events (%), and the y-axis lists individual adverse events. Each bar represents both the percentage and absolute number of cases (n). Abbreviations: COVID-19, Coronavirus disease 2019.

Twenty-seven significant safety signals for aducanumab adverse events were identified by meeting strict predefined criteria (Table 3). Amyloid related imaging abnormality-oedema/effusion (ARIA-E) related events were particularly prominent, with ARIA-E being the most frequent and strongly signaled event (n = 141, ROR: 53,538.3). This was followed by Amyloid related imaging abnormality-microhaemorrhages and haemosiderin deposits (ARIA-H) (n = 100). Other neurological events such as headache (n = 41) and cerebral hemorrhage (n = 27) also displayed strong signals. The distribution of individual adverse events (Figure 3) showed that ARIA-E was the most frequently reported event (12.88%, n = 141), followed by ARIA-H (9.13%, n = 100). Other significant neurological events included headache (3.74%, n = 41), confusional state (3.20%, n = 35), and cerebral hemorrhage (2.47%, n = 27). Cognitive-related events such as dementia Alzheimer’s type (1.92%, n = 21) and cognitive disorder (1.55%, n = 17) were also prominently reported.

**TABLE 3:** Multi-method disproportionality analysis of adverse events associated with aducanumab.

Preferred terms	Case number (n)	ROR (95% CI)	PRR (χ2)	IC(IC-2SD)	EBGM
ARIA-E	141	53538.3 (42177.8–67958.6)	46644.4 (3390009)	14.55 (6.83)	24044.0 (18942.0)
ARIA-H	100	38187.9 (29358.2–49673.2)	34700.5 (2041938)	14.32 (6.30)	20420.9 (15699.2)
Headache	41	3.77 (2.76–5.15)	3.66 (80.25)	1.87 (1.33)	3.66 (2.68)
Confusional state	35	12.42 (8.87–17.39)	12.05 (355.56)	3.59 (2.71)	12.05 (8.60)
Cerebral haemorrhage	27	42.86 (29.25–62.81)	41.83 (1075.76)	5.39 (3.54)	41.79 (28.52)
Fall	23	3.95 (2.61–5.97)	3.89 (49.58)	1.96 (1.20)	3.89 (2.57)
Superficial siderosis of CNS	22	35937.4 (20741.2–62267.2)	35215.4 (453124)	14.33 (3.80)	20598.1 (11888.2)
Dementia Alzheimer’s type	21	128.36 (83.30–197.79)	125.91 (2596.15)	6.97 (3.61)	125.60 (81.51)
Seizure	20	6.56 (4.21–10.21)	6.46 (92.50)	2.69 (1.72)	6.46 (4.15)
Amyloid related imaging abnormalities	17	17487.2 (10039.7–30459.3)	17215.7 (217258)	13.64 (3.40)	12781.6 (7338.13)
Cognitive disorder	13	16.05 (9.29–27.73)	15.87 (181.19)	3.99 (2.17)	15.86 (9.18)
Brain oedema	13	59.01 (34.14–101.99)	58.32 (731.68)	5.86 (2.74)	58.25 (33.70)
Memory impairment	12	4.89 (2.77–8.63)	4.84 (36.68)	2.28 (1.10)	4.84 (2.74)
Atrial fibrillation	11	6.35 (3.50–11.50)	6.29 (49.06)	2.65 (1.29)	6.29 (3.47)
Subarachnoid haemorrhage	9	48.94 (25.39–94.34)	48.54 (418.73)	5.60 (2.16)	48.50 (25.16)
Head injury	8	14.43 (7.20–28.93)	14.33 (99.25)	3.84 (1.56)	14.33 (7.15)
Cerebral microhaemorrhage	7	1493.95 (702.77–3175.87)	1484.41 (10075.5)	10.49 (1.95)	1441.32 (678.01)
Transient ischaemic attack	5	8.02 (3.33–19.31)	7.99 (30.57)	3.00 (0.70)	7.99 (3.32)
Mental status changes	5	10.00 (4.15–24.07)	9.96 (40.30)	3.32 (0.82)	9.95 (4.13)
Aphasia	4	7.20 (2.70–19.22)	7.18 (21.28)	2.84 (0.39)	7.18 (2.69)
Disorientation	4	5.49 (2.06–14.66)	5.48 (14.64)	2.45 (0.24)	5.47 (2.05)
Subdural haematoma	3	11.44 (3.68–35.52)	11.41 (28.48)	3.51 (0.22)	11.40 (3.67)
Skin cancer	3	7.94 (2.56–24.67)	7.92 (18.15)	2.99 (0.09)	7.92 (2.55)
Posterior reversible encephalopathy syndrome	3	17.14 (5.52–53.22)	17.09 (45.44)	4.09 (0.32)	17.09 (5.50)
Cerebral infarction	3	6.76 (2.18–20.98)	6.74 (14.67)	2.75 (0.02)	6.74 (2.17)
Haemorrhage intracranial	3	10.71 (3.45–33.27)	10.69 (26.34)	3.42 (0.20)	10.68 (3.44)
Ischaemic stroke	3	9.32 (3.00–28.93)	9.29 (22.20)	3.22 (0.15)	9.29 (2.99)

Abbreviations: ARIA-E, Amyloid related imaging abnormality-oedema/effusion; ARIA-H, Amyloid related imaging abnormality-microhaemorrhages and haemosiderin deposits; CI, confidence interval; CNS, central nervous system; EBGM, empirical bayes geometric mean; IC, information component; IC-2SD, information component minus two standard deviations; PRR, proportional reporting ratio; ROR, reporting odds ratio; SD, standard deviation.

### 3.5 Temporal analysis of safety signals

**FIGURE 4:**
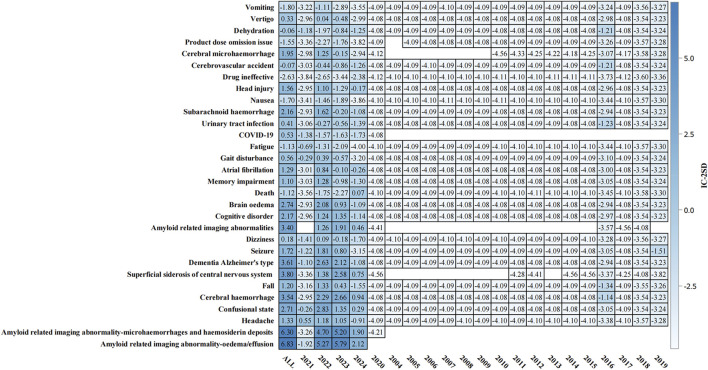
Temporal analysis of adverse events associated with aducanumab: Information component signal detection from 2004–2024. The heat map presents the information component (IC) analysis of adverse events associated with aducanumab across different time periods (2004–2024). The color intensity represents IC-2SD values, with darker red indicating stronger positive signals. The y-axis lists preferred terms (PT), and the x-axis shows the temporal distribution. Abbreviations: COVID-19, Coronavirus disease 2019; IC, Information component; IC-2SD, Information component minus 2 standard deviations.

The temporal analysis from 2004 to 2024 (Figure 4) demonstrated progressive accumulation of safety signals, particularly for ARIA-related events. The heat map visualization showed that ARIA-E and ARIA-H maintained consistently strong positive signals (IC-2SD values of 6.83 and 6.30, respectively) throughout the surveillance period. The intensity of safety signals for neurological events showed notable increases in recent years, particularly during 2022–2023.

### 3.6 Stratified analysis

**FIGURE 5:**
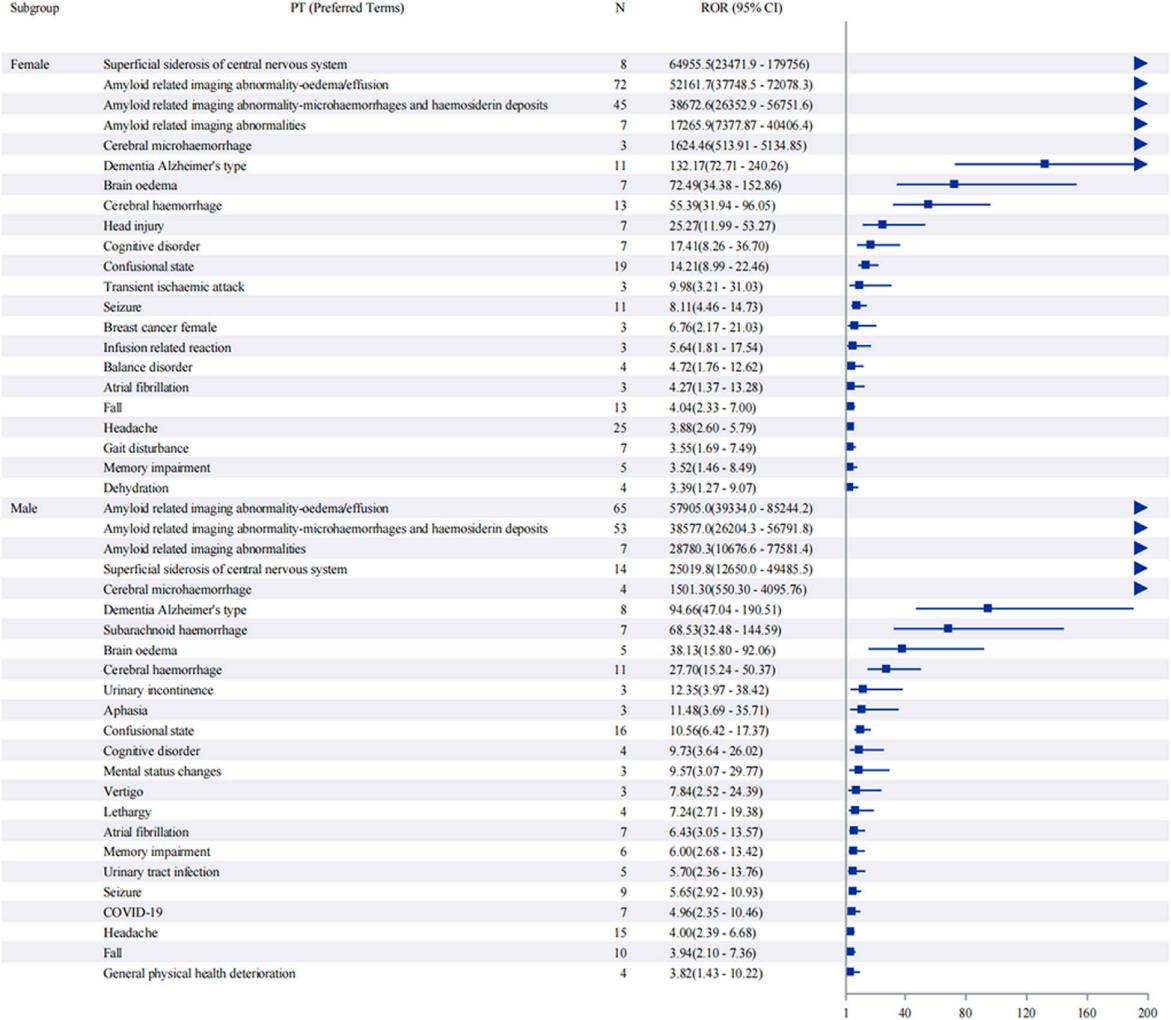
Sex-stratified analysis of adverse events associated with aducanumab: A reporting odds ratio analysis. The report of odds ratio (ROR) analysis of adverse events stratified by sex for aducanumab treatment. The forest plot displays preferred terms (PT) on the y-axis and corresponding ROR values with 95% confidence intervals (CI) on the x-axis. Notable signals include ARIA-E, ARIA-H, and other neurological events. The analysis demonstrates similar safety profiles between male and female subgroups, with ARIA-related events showing the highest reporting odds in both sexes. Abbreviations: ARIA, Amyloid related imaging abnormalities; ARIA-E, Amyloid related imaging abnormality-oedema/effusion; ARIA-H, Amyloid related imaging abnormality-microhaemorrhages and haemosiderin deposits; CI, Confidence interval; COVID-19, Coronavirus disease 2019; PT, Preferred terms; ROR, Reporting odds ratio; UTI, Urinary tract infection.

**FIGURE 6:**
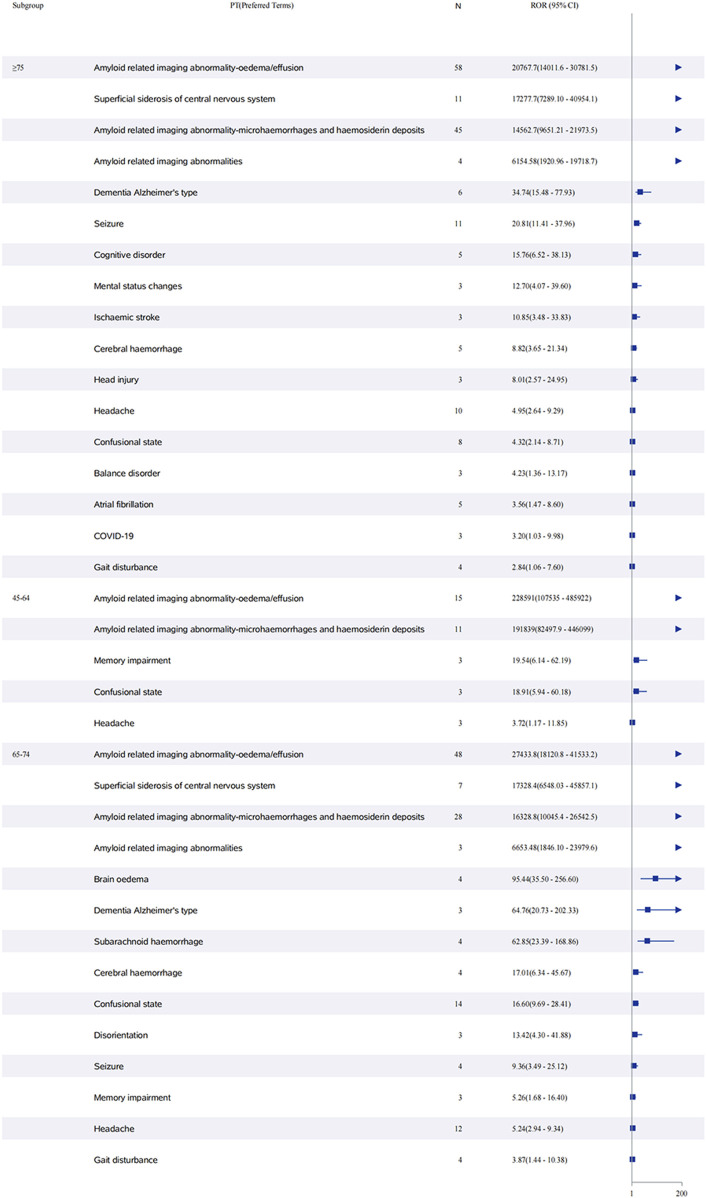
Age-stratified analysis of adverse events associated with aducanumab: A reporting odds ratio analysis in three age groups. The report of odds ratio (ROR) analysis of adverse events stratified by age groups (45–64, 65–74, and ≥75 years) for aducanumab treatment. The forest plot displays preferred terms (PT) on the y-axis and corresponding ROR values with 95% confidence intervals (CI) on the x-axis. ARIA-related events showed the highest reporting odds across all age groups. Other significant adverse events include cognitive disorders, neurological symptoms, and cerebrovascular events, with varying frequencies among different age groups. Abbreviations: ARIA, Amyloid related imaging abnormalities; ARIA-E, Amyloid related imaging abnormality-oedema/effusion; ARIA-H, Amyloid related imaging abnormality-microhaemorrhages and haemosiderin deposits; CI, Confidence interval; COVID-19, Coronavirus disease 2019; PT, Preferred terms; ROR, Reporting odds ratio.

Sex-stratified analysis (Figure 5) revealed comparable safety profiles between males and females. In females, ARIA-E showed the strongest signal (ROR: 52,161.7, 95% CI: 37,748.5–72,078.3), followed by ARIA-H (ROR: 38,672.6, 95% CI: 26,352.9–56,751.6). Males demonstrated similar patterns, with ARIA-E (ROR: 57,905.0, 95% CI: 39,334.0–85,244.2) and ARIA-H (ROR: 38,577.0, 95% CI: 26,204.3–56,791.8) being the most significant events. The age-stratified analysis (Figure 6) across three groups (45–64, 65–74, and ≥75 years) showed that ARIA-related events maintained strong signals across all age groups, with the highest reporting odds in the ≥75 years group. While cognitive disorders and neurological symptoms showed varying reporting frequencies among different age groups, with more reports from older patients, these findings reflect only reporting patterns rather than true incidence rates.

### 3.7 Time-to-onset analysis and comprehensive signal analysis

**FIGURE 7:**
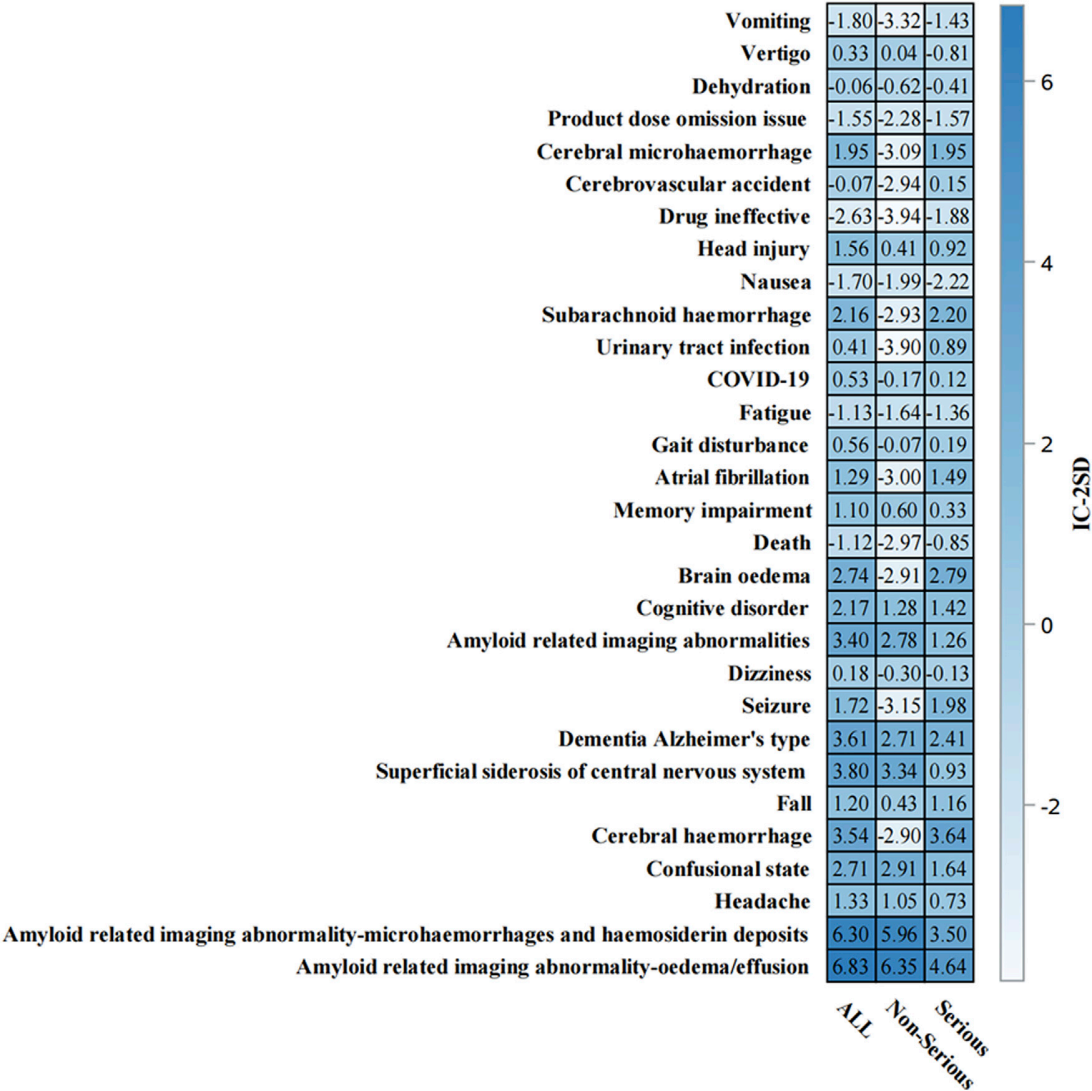
Information component analysis heat map of aducanumab-associated adverse events from. Adverse events are listed on the y-axis, while the x-axis displays IC-2SD values for three categories: ALL, Non-Serious, and Serious events. The blue intensity represents the signal strength. Abbreviations: COVID-19, Coronavirus disease 2019; IC, Information component; SD, Standard deviation.

Time-to-onset analysis (Supplementary Figure S1) revealed a median time to event of 146.0 days (IQR: 80.0–195.0 days). The cumulative distribution showed a relatively steady increase in event occurrence over time, with most events occurring within the first 360 days of treatment initiation. The disproportionality analysis (Supplementary Figures S2) confirmed the robustness of safety signals, particularly for ARIA-related events. ARIA-E and ARIA-H showed the highest safety signals (IC-2SD: ALL = 6.83 and 6.30, respectively) (Figure 7). Other significant neurological events included superficial siderosis, cerebral hemorrhage, and confusional state (Figure 7).

## 4 Discussions

This comprehensive pharmacovigilance analysis of the Food Drug Administration Adverse Event Reporting System (FAERS) database has provided crucial insights into the safety profile of aducanumab in real-world clinical practice. As the first anti-amyloid β monoclonal antibody to receive FDA accelerated approval for Alzheimer’s disease treatment (Cummings et al., 2021), understanding aducanumab’s safety characteristics is paramount for optimal clinical implementation and patient care management.

The adverse events identified in our analysis demonstrate a clear correlation with the drug’s mechanism of action. Aducanumab functions by selectively binding to and facilitating the clearance of amyloid β plaques in the mice brain, representing a significant advancement in disease-modifying therapies for Alzheimer’s disease (Sevigny et al., 2016). The most prominent safety signals observed were Amyloid-Related Imaging Abnormalities (ARIA), specifically ARIA-E (edema) and Amyloid related imaging abnormality-microhaemorrhages and haemosiderin deposits (ARIA-H), which are mechanistically linked to the drug’s effects on cerebral vasculature during amyloid protein clearance (Hampel et al., 2023; Doran and Sawyer, 2024). While these findings align with earlier clinical trial data, our study provides robust confirmation through larger-scale real-world evidence, offering clinicians valuable insights into the practical aspects of aducanumab administration.

A particularly noteworthy finding is the sex-neutral distribution of adverse events, suggesting that aducanumab’s safety profile remains consistent across sex (Schneider, 2020; Choi et al., 2023). This observation contrasts with some previous studies of neurological therapeutics where sex-specific differences were noted, potentially simplifying clinical decision-making processes. Higher adverse event rates were observed in patients ≥75 years, consistent with the typical age distribution of dementia, necessitating closer monitoring in this population (Liew, 2020). The temporal analysis demonstrated that most adverse events manifested within 360 days of treatment initiation, with a median onset time of 146 days, establishing a critical monitoring window for clinical surveillance. This temporal pattern provides clinicians with a clear framework for patient monitoring and risk management strategies.

Our study’s methodological strength lies in its novel application of four complementary disproportionality analysis methods, substantially enhancing the reliability of signal detection. This multi-modal analytical approach, unprecedented in previous aducanumab safety studies, provides a more comprehensive understanding of the drug’s safety profile (Wang et al., 2024). Recent evidence suggests that aducanumab, as a disease-modifying therapeutic agent, may offer enhanced efficacy when administered early in the disease course (Cummings et al., 2023; Ameen et al., 2024). This observation underscores the importance of carefully balancing therapeutic benefits against potential risks, particularly in early-stage Alzheimer’s disease patients. Despite ongoing debates surrounding the amyloid hypothesis, compelling evidence from human genetics, biochemical analyses, histopathological examinations, and animal models strongly supports the pivotal role of β-amyloid in Alzheimer’s disease pathogenesis (Musiek et al., 2021). Notably, extensive studies in transgenic mouse models have demonstrated that aducanumab penetrates the blood-brain barrier, selectively binds to parenchymal Aβ, and substantially reduces both soluble and insoluble Aβ levels in a dose-dependent manner (Sevigny et al., 2016). These preclinical findings align remarkably well with clinical observations, lending further credence to the therapeutic potential of this agent. Proteomic analyses have revealed significant modulation of several key proteins associated with mitochondrial function and metabolism in aducanumab-treated mice (Bastrup et al., 2021), offering fresh insights into the mechanism of action. Furthermore, three clinical studies have confirmed that aducanumab (3–10 mg/kg) significantly reduced amyloid plaque deposition as measured by PET imaging over a 1-year period compared to placebo (Day et al., 2022). This multilayered experimental evidence, particularly the marked effects observed in animal models, provides a robust scientific foundation for the clinical application of aducanumab.

The geographic distribution of adverse event reports, predominantly from the United States, reflects the drug’s current approval status and usage patterns. This concentration of data from a single healthcare system provides consistency in reporting standards but also highlights the need for broader international safety surveillance as global adoption increases (Fang et al., 2014). Our large sample size and rigorous statistical methodology provide compelling safety signal evidence, despite the inherent limitations of FAERS data, including reporting bias and challenges in establishing causality.

Future research directions should focus on collecting long-term safety data and examining treatment experiences across diverse populations (Dickson et al., 2023). Particular attention should be paid to understanding the impact of genetic factors, such as APOE ε4 carrier status, on safety outcomes (Raulin et al., 2022; Arafah et al., 2023; Tripathi et al., 2024). This could be implemented through routine APOE genotyping using established cost-effective methods, such as PCR-based techniques or mass spectrometry (Calero et al., 2018), which can be integrated into standard clinical workflows. While current insurance coverage for APOE testing varies among payers (Arias et al., 2021), emerging evidence supporting its role in treatment decision-making may justify broader coverage policies (Blasco and Roberts, 2023). Additionally, investigation of potential drug interactions and the effects of concurrent medications commonly prescribed to elderly patients warrants further study.

The safety signals identified through our analysis, particularly ARIA-related events, warrant careful consideration but should be viewed within the context of the drug’s potential therapeutic benefits (Vaz et al., 2022). Our findings suggest that with appropriate patient selection and monitoring protocols, aducanumab maintains a manageable safety profile. This research provides valuable insights for risk management in clinical practice and supports the optimization of personalized treatment strategies.

In conclusion, while ARIA and other adverse events require vigilant monitoring, aducanumab emerges as a viable therapeutic option when accompanied by appropriate patient screening and monitoring protocols. The establishment of clear safety patterns and risk factors through this analysis contributes significantly to the evolving landscape of Alzheimer’s disease treatment, providing clinicians with essential data for informed decision-making and patient care optimization. As our understanding of the drug’s long-term safety profile continues to evolve, ongoing pharmacovigilance efforts will remain crucial in ensuring optimal therapeutic outcomes for patients with Alzheimer’s disease.

## Data Availability

The original contributions presented in the study are included in the article/Supplementary Material, further inquiries can be directed to the corresponding author.

## Supplementary material

SUPPLEMENTARY FIGURE S1 Time-to-onset analysis of adverse events associated with aducanumab: A cumulative distribution analysis. The figure presents a cumulative time-to-onset analysis of adverse events associated with aducanumab treatment. The x-axis shows the time to event in days (0–360), and the y-axis displays the cumulative percentage of events (0–100%). The median time to event and interquartile range (IQR) are indicated. Abbreviations: IQR, Interquartile range.

SUPPLEMENTARY FIGURE S2 Adverse events analysis of aducanumab: Reporting odds ratio analysis of preferred terms. The reporting odds ratio (ROR) analysis of adverse events associated with aducanumab. The x-axis shows ROR values with 95% confidence intervals (CI), and the y-axis lists Preferred terms (PT). The analysis reveals significant signals for ARIA-E, ARIA-H, and other neurological events. Abbreviations: ARIA, Amyloid related imaging abnormalities; ARIA-E, Amyloid related imaging abnormality-oedema/effusion; ARIA-H, Amyloid related imaging abnormality-microhaemorrhages and haemosiderin deposits; CI, Confidence interval; PT, Preferred terms; ROR, Reporting odds ratio.
