# Supplementary figures and images for: A real-world safety surveillance study of aducanumab through the FDA adverse event reporting system
Source: Front Pharmacol. 2025 Mar 13;16:1522058. doi: 10.3389/fphar.2025.1522058 (PMC11960500; doi:10.3389/fphar.2025.1522058)

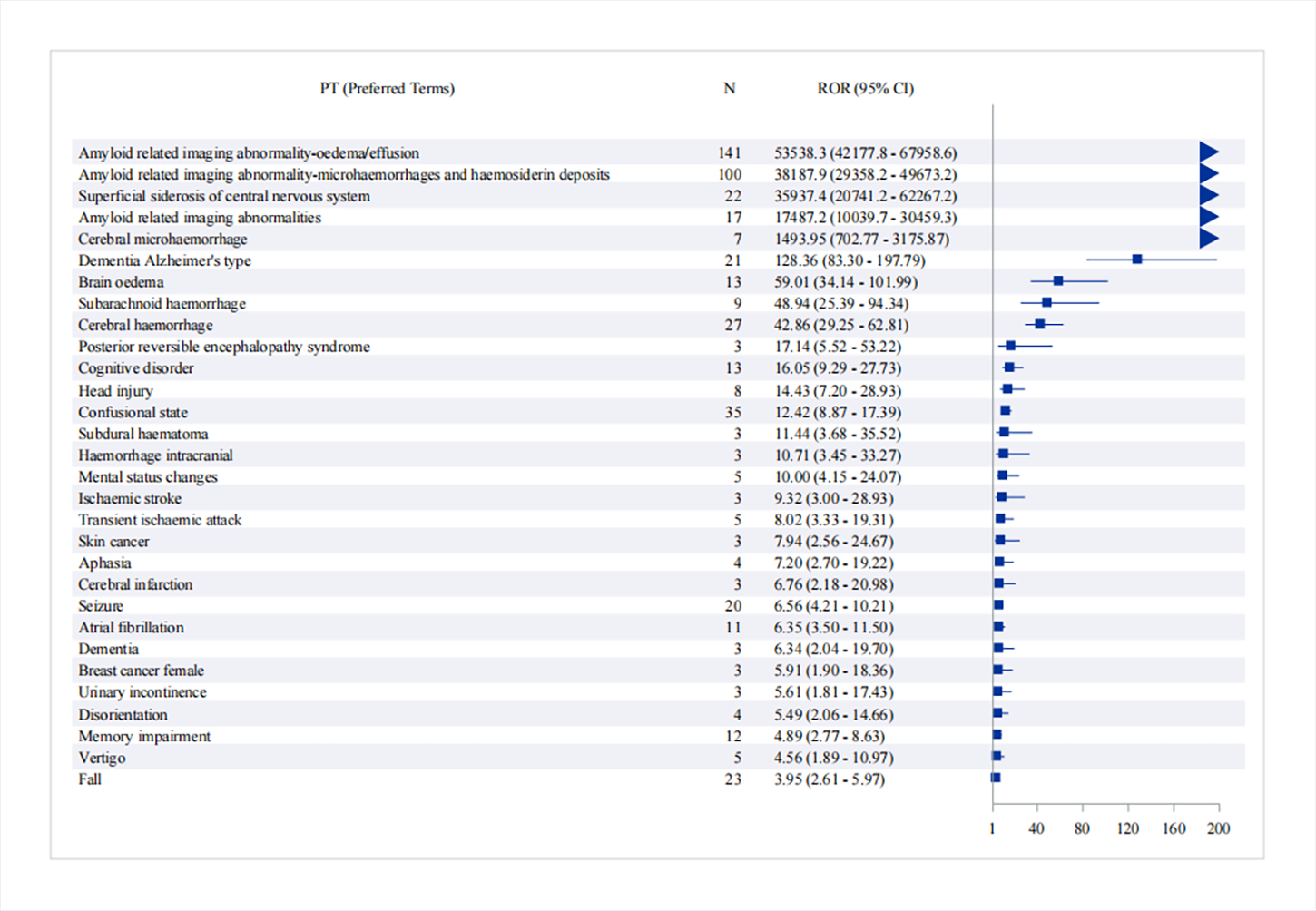

Supplement: Supplementary file 1 [file Image2.tif]

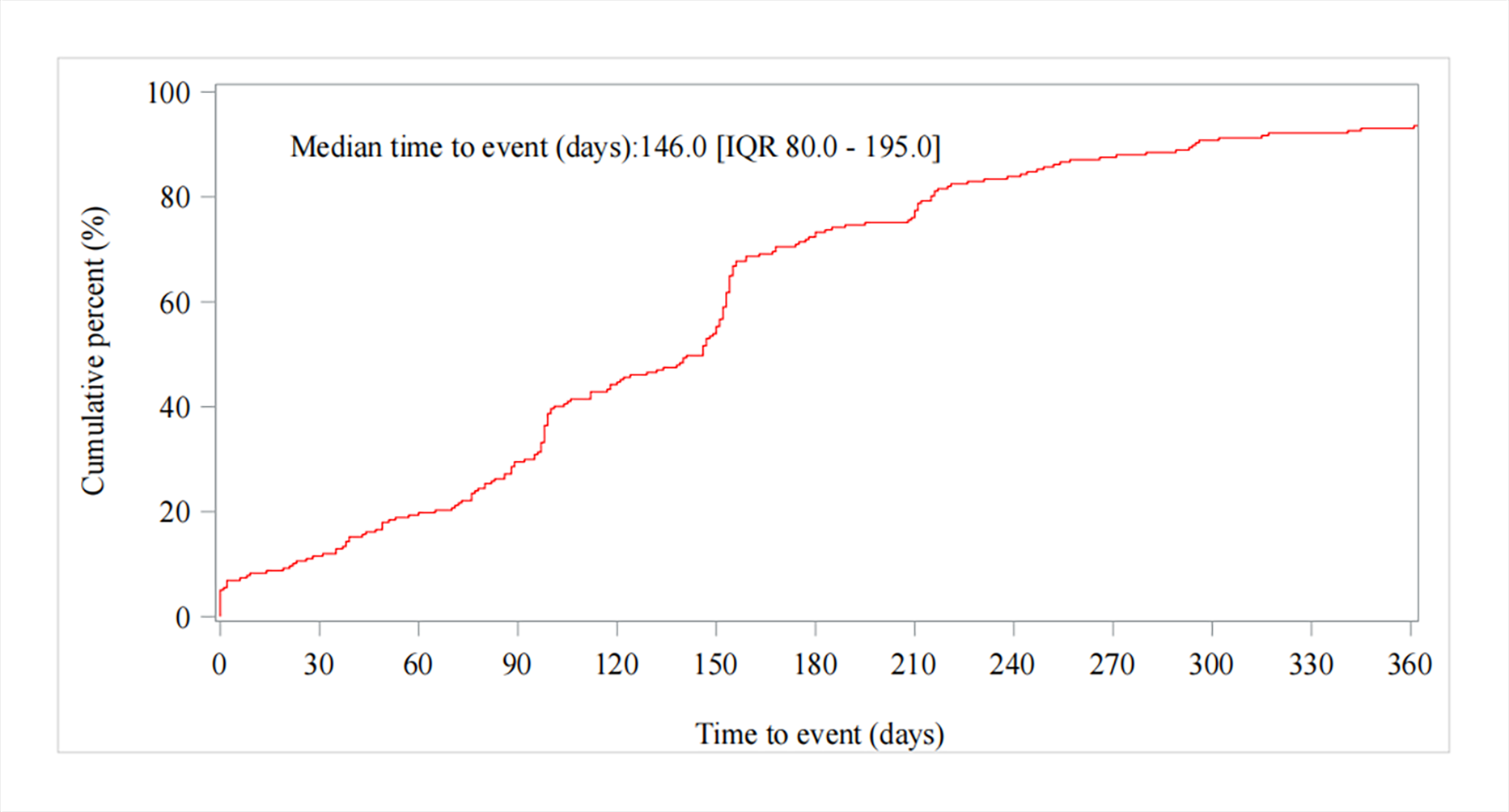

Supplement: Supplementary file 2 [file Image1.tif]
